# That Boston Resolution

**Published:** 1906-09

**Authors:** 


THAT BOSTON RESOLUTION.—It is a pity that faith in the sincerity of an organization as representative and effective as the American Medical Association should be undermined by a blank refusal at the Boston meeting to have the affairs of the Association carefully audited by a committee composed of men of unquestioned fairness and integrity. In reality it should have been unnecessary to pass such a resolution, for in an absolutely democratic institution as this body purports to be there should be made and published annually a detailed report of the affairs of the Association and the methods and conduct of its employes.

Thousands who have hitherto had the utmost confidence in the present regime will now, whether justly or unjustly, have suspicions aroused which will seriously affect the efficiency of the organization, and it is to be seriously hoped for the premanence and integrity of this magnificently planned institution that all inquiries may be openly and frankly answered. Secrecy in the affairs of any democratic body is not only injudicious, but it fosters an increasing element of discord which will ultimately lead to disruption. The action of the Boston meeting is therefore to be both deplored and condemned.—*Southern Med. and Surgery.*

